# Honokiol Eliminates Glioma/Glioblastoma Stem Cell-Like Cells via JAK-STAT3 Signaling and Inhibits Tumor Progression by Targeting Epidermal Growth Factor Receptor

**DOI:** 10.3390/cancers11010022

**Published:** 2018-12-26

**Authors:** Yipu Fan, Weikang Xue, Melitta Schachner, Weijiang Zhao

**Affiliations:** 1 Center for Neuroscience, Shantou University Medical College, 22 Xin Ling Road, Shantou, Guangdong 515041, China; yipu_fan@163.com (Y.F.); weikangxue1994@163.com (W.X.); schachner@stu.edu.cn (M.S.); 2 Keck Center for Collaborative Neuroscience and Department of Cell Biology and Neuroscience, Rutgers University, 604 Allison Road, Piscataway, NJ 08854, USA

**Keywords:** honokiol, glioma/glioblastoma, glioma stem cell, EGFR, apoptosis, metastasis

## Abstract

Malignant gliomas are the most aggressive forms of brain tumors; whose metastasis and recurrence contribute to high rates of morbidity and mortality. Glioma stem cell-like cells are a subpopulation of tumor-initiating cells responsible for glioma tumorigenesis, metastasis, recurrence and resistance to therapy. Epidermal growth factor receptor (EGFR) has been reported to be dysregulated in most cancers, including gliomas and its functions are closely linked to initiating tumor metastasis and a very poor prognosis. In search for compounds that may reduce the tumorigenic potential of gliomas/glioblastomas honokiol attracted our attention. Honokiol, purified from the bark of traditional Chinese herbal medicine Magnolia species, is beneficial in vitro and in animal models via a variety of pharmacological effects, including anti-inflammatory, anti-angiogenetic, anti-arrhythmic and antioxidant activities, as well as anti-proliferative and proapoptotic effects in a wide range of human cancer cells. However, its effects on glioma cells are unknown. Here, we used different concentrations of honokiol in treating U251 and U-87 MG human glioma/glioblastoma cells in cell culture. Results showed that honokiol inhibited glioma cell viability and colony formation and promoted apoptosis. It also inhibited glioma cell migration/proliferation and invasion. In addition, honokiol promoted apoptosis and reduced Bcl-2 expression, accompanied by increase in Bax expression. Honokiol reduced expression of EGFR, CD133 and Nestin. Moreover, honokiol inhibited the activation of both AKT and ERK signaling pathways, increased active caspase-3 level and reduced phosphorylation of STAT3. U-87 MG xenografts in nude mice and in immunotolerant zebrafish yolk sac showed that honokiol inhibits tumor growth and metastasis. Altogether, results indicate that honokiol reduces tumorigenic potentials, suggesting hopes for honokiol to be useful in the clinical management of glioma/glioblastoma.

## 1. Introduction

Glioblastoma (GBM) is a highly lethal brain tumor that is resistant to most therapeutic means currently available. It accounts for 70% of human primary malignant brain tumors [1]. The current treatment for GBM patients mostly depends on surgical resection in combination with radiotherapy and chemotherapy [2]. Despite significant advances in cancer treatment, little improvement has been achieved in the prognosis of GBM patients over decades [3].

Cancer stem cells represent a subpopulation of tumor cells with enhanced clonogenic and tumorigenic potentials within a tumor [4]. A variety of tumors have been reported to contain a cell population with stem cell-like properties, including self-renewal and differentiation [5]. A number of tumor types, including gliomas, have shown the existence of a unique population of tumor/glioma initiating cells [4], which were initially isolated from human gliomas for isolating neural stem cells. These are biologically characterized as embryonic and/or tissue-restricted stem cells with self-renewal capacity and ability to differentiate along tissue-specific lineage [6]. Such stem cells are believed to cause relapse after tumor resection, thereby contributing to treatment resistance [7]. Glioma stem cells can be enriched by cell sorting via cell surface proteins such as CD44 and CD133 [8] and are thus amenable to investigations focusing on factors that influence their functions.

Among the molecules contributing to tumorigenesis, epidermal growth factor receptor (EGFR) has been recognized to induce neoplastic transformation in the presence of ligands and its overexpression is characteristic of several cancer cell lines [9]. Specifically, in human gliomas EGFR is not only overexpressed but also occurs in several mutations [10]. It is activated by mitogenic ligands, including epidermal growth factor (EGF), heparin-binding epidermal growth factor (HB-EGF), transforming growth factor-α (TGF-α), amphiregulin, epiregulin and betacellulin [11]. Ligand binding leads to homo- or heterodimerization of EGFR with other ligand-bound ErbB receptors, thus transmitting mitogenic signals to downstream cell survival and proliferation involved signaling cascades, including phosphatidylinositol 3-kinase (PI3K)/Akt, mitogen-activated protein kinase (MAPK) and signal transducer and activator of transcription 3 (STAT3) [9].

Honokiol, used in traditional Chinese medicine, is a natural compound extracted from the bark of the magnolia tree. Studies have demonstrated a wide range of its pharmaceutical effects, including anti-inflammatory, anti-microbial and antioxidant activities as well as protection against hepatotoxicity, neurotoxicity, thrombosis and angiopathy [12]. Honokiol has also been reported to target EGFR signaling via STAT3 in the treatment of head/neck cancer, thus enhancing the effects of EGFR-targeting therapies [13]. It affects tumor cell growth and survival by inhibition of the nuclear factor kappa B (NFκB) signaling pathway and altering other molecular targets in several cancer models [14].

Here, we show that honokiol inhibits glioma cell viability and migration/invasion and induces apoptosis of human glioma/glioblastoma cell lines. We also show that honokiol reduces EGFR expression and down-regulates JAK/STAT3 signaling pathways. In addition, honokiol decreases the number of CD133 positive stem cells and promotes elimination of a glioma stem cell subpopulation. *In vivo*, honokiol profoundly suppresses glioblastoma xenograft growth in nude mice and in zebrafish yolk sack.

## 2. Results

### 2.1. Honokiol Inhibits Glioma/Glioblatoma Cell Viability

**Figure 1:**
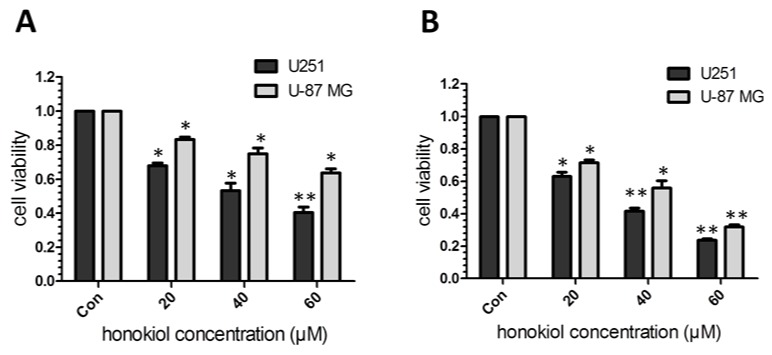
Effect of honokiol on glioma/glioblastoma cell viability. U251 human glioma and U-87 MG human glioblastoma cells were treated with different concentrations of honokiol for 24 (**A**) and 48 h (**B**). MTT assay was used to assess cell viability. Data are presented as means ± SEM from 3 independent experiments. IC_50_ values for U251 and U-87 MG at 24 h were 54 μM and 62.5 μM, respectively. * *p* < 0.05 and ** *p* < 0.01 vs. vehicle control (one-way ANOVA with Tukey’s *post-hoc* test).

At concentrations of honokiol ranging from 0 to 60 µM, cell viability was dose-dependently reduced for U251 and U-87 MG cells compared with the vehicle control after treatment for 24 and 49 h (Figure 1A,B).

### 2.2. Honokiol Inhibits Cell Migration/Proliferation and Invasion

**Figure 2:**
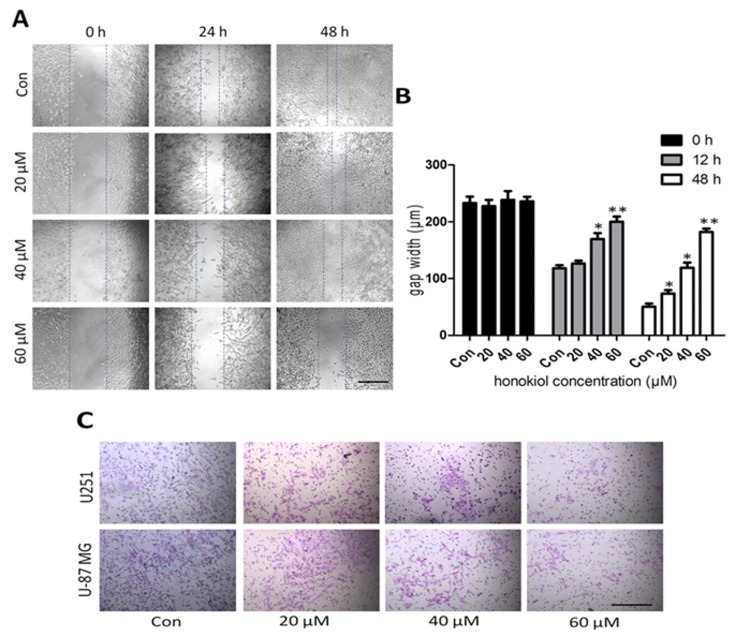

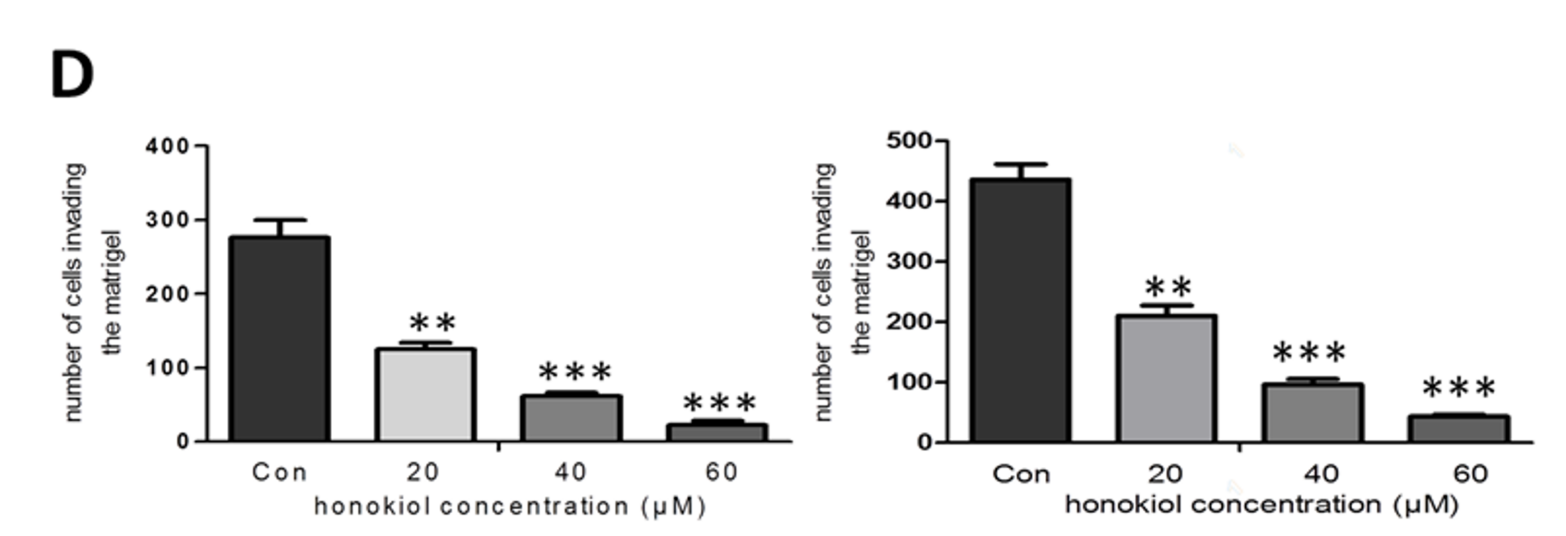
Honokiol reduces U251 and U-87 MG cell migration/proliferation and invasion. (**A**) Representative images captured under a phase contrast microscope after 24 h and 48 h of treatment with different concentrations of honokiol. The vertical lines indicated the wound edge. Scale bar: 200 μm. (**B**) Shown are the average gap widths, as determined by Image J. (**C**). Representative images of trans-migrated glioma/glioblastoma cells stained with crystal violet. Scale bar: 200 μm. (**D**) Quantification of transmigrated cells. Data are presented as means ± SEM from 3 independent experiments. * *p* < 0.05, ** *p* < 0.01 and *** *p* < 0.001 vs. vehicle control group (one-way ANOVA with Tukey’s *post-hoc* test).

Scratch assay with U-87 MG cells (Figure 2A,B) and transwell cell invasion assay with U251 and U-87 cells (Figure 2C,D) were used to evaluate the effects of honokiol on glioma cell migration/proliferation and invasion, respectively. No difference in gap widths was detected at 0 h after scratching (Figure 2B). After 24 and 48 h of incubation, 20, 40 and 60 μM of honokiol impeded gap closure of U-87 MG cells, with the most effective inhibition observed at 60 μM for both incubation times (Figure 2B). As was indicated by the number of cells having migrated to the underside of the transwell chamber, honokiol dose-dependently reduced the invasion ability of both cell lines when compared to vehicle control (Figure 2C,D). These results indicate that honokiol reduces cell migration/proliferation and invasion abilities.

### 2.3. Honokiol Inhibits Colony Formation

**Figure 3:**
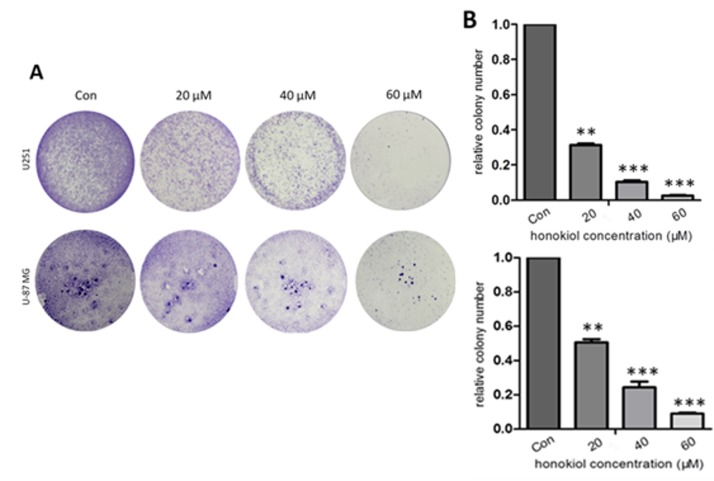
Quantification of colony formation. Representative images from 3 independent experiments are shown in (**A**). As indicated by the relative level of colony formation, honokiol inhibits colony formation of U251 and U-87 MG cells (**B**). Data are presented as means ± SEM from 3 independent experiments. ** *p* < 0.01 and *** *p* < 0.001 vs. vehicle control group (one-way ANOVA with Tukey’s *post-hoc* test).

In the colony-formation assay honokiol suppresses colony formation in a dose-dependent manner when compared with the vehicle control (Figure 3A,B).

### 2.4. Honokiol Promotes Apoptosis

**Figure 4:**
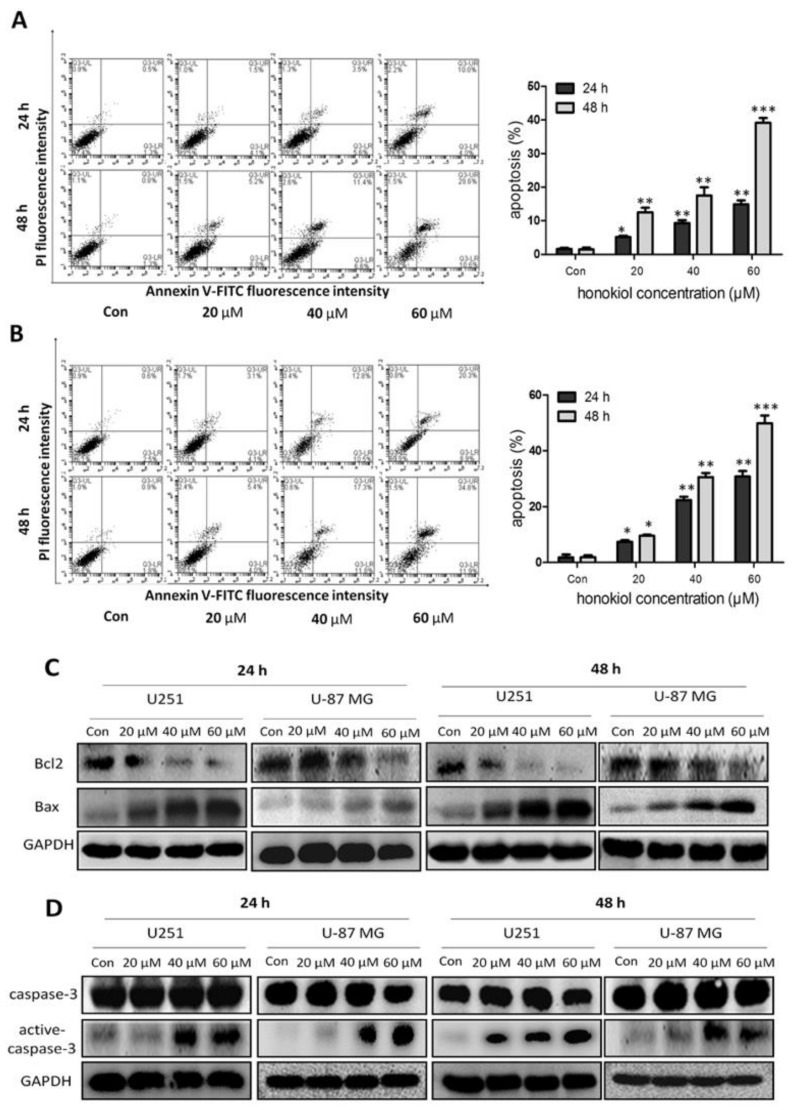
Honokiol promotes apoptosis. (**A**,**B**) Two cell lines were treated with 0, 20, 40, 60 µM honokiol for 24 h and 48 h and thereafter stained with Annexin V-EGFP/PI. The percentage of apoptotic cells was determined using flow cytometry. Data are presented as means ± SEM from 3 independent experiments. (* *p* < 0.05, ** *p* < 0.01 and *** *p* < 0.001 vs. vehicle control group) (**C**) Western blot analysis of Bcl-2 and Bax expression in U251 and U-87 MG cells treated with 0, 20, 40, 60 µM honokiol for 24 and 48 h. (**D**) Western blot analysis of full length and cleaved caspase-3 in glioma/glioblastoma cells treated with 0, 20, 40 and 60 µM honokiol for 24 h and 48 h.

In the Annexin V-EGFP/PI apoptosis assay, honokiol induced apoptosis in both U251 (Figure 4A) and U-87 MG cells (Figure 4B) when compared to the vehicle control. Honokiol dose-dependently reduced Bcl-2 protein level, while increasing Bax level in both lines after 24 and 48 h incubation (Figure 4C). In addition, honokiol dose-dependently promoted the cleavage of caspase-3 at 24 and 48 h incubation times (Figure 4D). These findings show the apoptosis promoting potential of honokiol.

### 2.5. Honokiol Inhibits Erk and Akt Signaling Pathways

**Figure 5:**
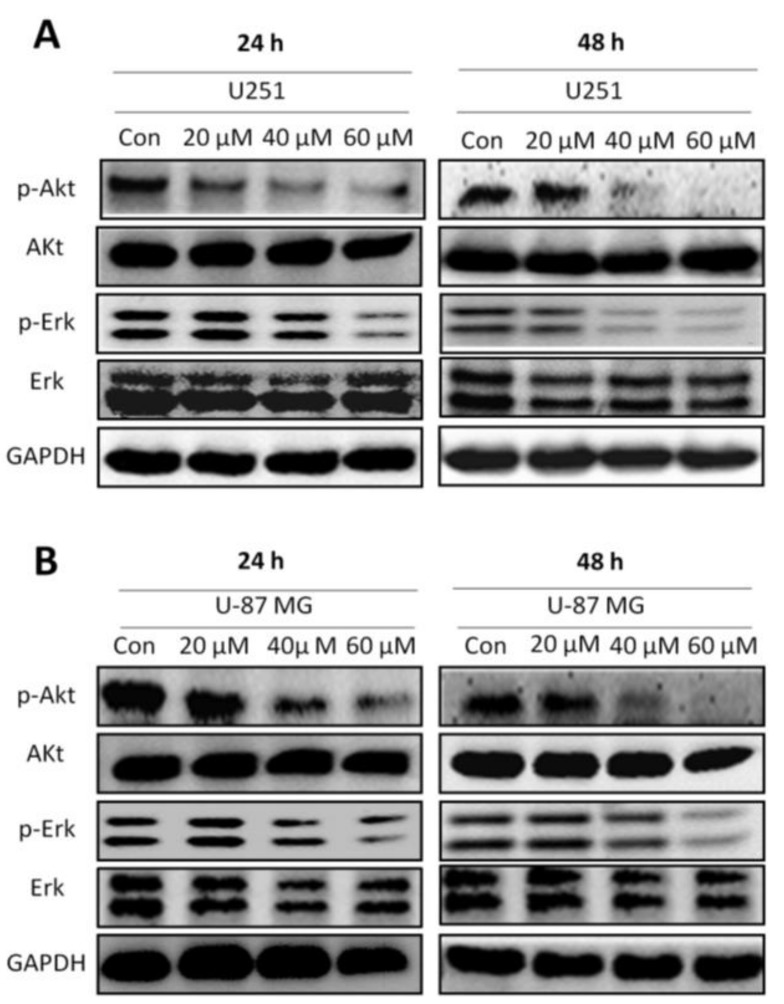
Honokiol inhibits the activation of Erk and Akt signaling pathways. (**A**) p-Akt and p-Erk protein levels in U251 cells were determined by western blot analysis after treatment with different concentrations of honokiol for 24 and 48 h. (**B**) p-Akt and p-Erk protein levels in U-87 MG cells were determined by western blot analysis after treatment with different concentrations of honokiol for 24 and 48 h.

Phosphorylation of Erk and Ark was measured by western blot analysis after incubation with honokiol (20, 40 and 60 μM) for 24 and 48 h, showing that p-Akt levels were profoundly reduced after treatment with honokiol at 40 and 60 μM in both cell lines at both 24 h and 48 h incubation times (Figure 5A,B). Similarly, U251 cells treated with 60 μM honokiol showed a reduced p-Erk level at 24 h, with a considerable reduction in p-Erk level at 48 h incubation time at doses of 40 and 60 μM (Figure 5A). In contrast, p-Erk was strongly reduced only in U-87 MG cells treated with 60 μM honokiol at both incubation time points (Figure 5B).

### 2.6. Honokiol Inhibits Expression of MMP2 and MMP9 and EMT-Related Molecules

**Figure 6:**
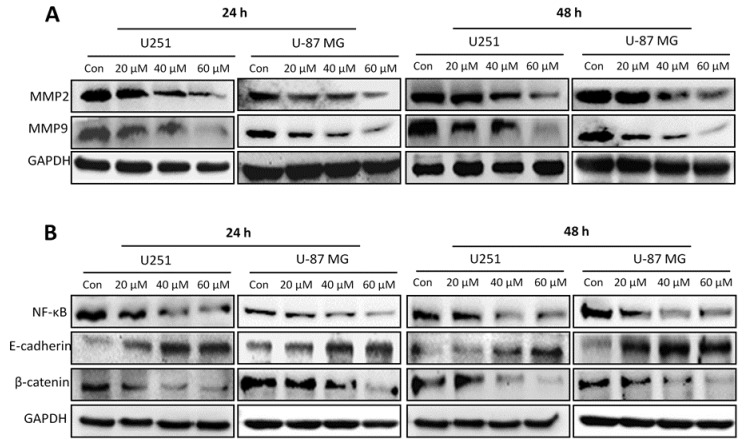
Honokiol inhibits MMP2 and MMP9 expression, as well as NFκB, E-cadherin and β-catenin expression in both cell lines. (**A**) MMP2 and MMP9 protein levels were determined by western blot analysis in U251 and U-87 MG cells after treatment with different concentrations of honokiol for 24 and 48 h. (**B**) NFκB, E-cadherin and β-catenin protein levels were determined by western blot analysis in U251 and U-87 MG cells after treatment with different concentrations of honokiol for 24 and 48 h.

Both MMP2 and MMP9 have been reported to function in tumor cell invasion, thus leading to metastasis. Honokiol dose-dependently reduced the protein levels of MMP2 and MMP9 in both cell lines (Figure 6A). In addition, honokiol dose-dependently reduced the protein levels of NF-κB and its signaling pathway molecules, including E-cadherin and β-catenin in both cell lines (Figure 6B).

### 2.7. Honokiol Inhibits the STAT3 Signaling Pathway via EGFR

**Figure 7:**
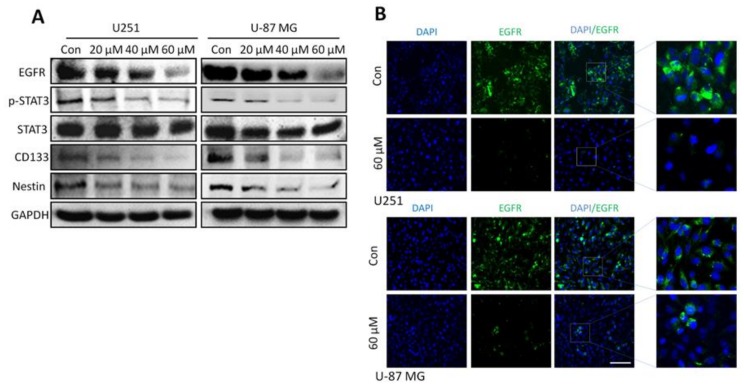
Honokiol inhibits EGFR signaling in glioma cells. (**A**) U251 and U-87 MG cells were treated with 0, 20, 40 and 60 µM honokiol for 48 h and EGFR, p-STAT3, CD133 and Nestin levels were determined by western blot analysis. (**B**) U251 and U-87 MG cells were treated with 0 and 60 µM honokiol for 48 h. Shown are photographs demonstrating cells stained with the EGFR (green) by fluorescence microscopy. Nuclei were counterstained with DAPI (blue). Scale bar: 200 μm.

The EGFR signaling pathway, including activation of STAT3 and increased expression of STAT3 target genes, plays an important role in glioma development. Honokiol dose-dependently inhibits EGFR expression and down-regulates STAT3 phosphorylation at 24 to 48 h of incubation times (Figure 7A). This down-regulation was also observed for CD133 and Nestin (Figure 7A). The effects on EGFR expression were confirmed by immunofluorescence staining, showing reduced fluorescence intensity for EGFR labeling in response to 60 μM of honokiol at 48 h incubation time (Figure 7B).

### 2.8. Honokiol Inhibits Stem Cell Spheroid Formation

**Figure 8:**
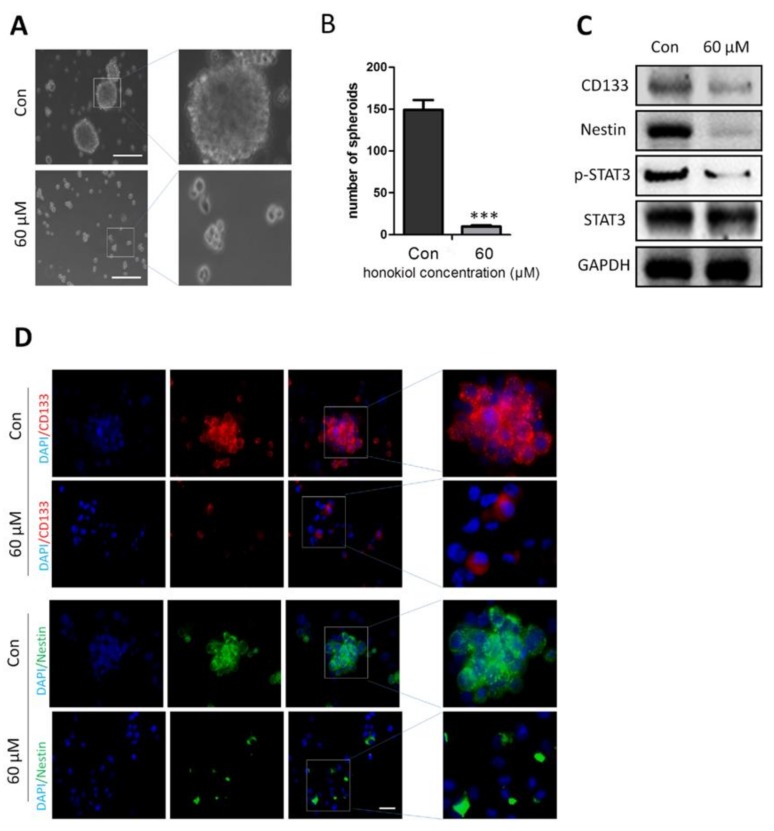
Honkiol inhibits spheroid formation of glioblastoma cells. (**A**–**D**) CD133^+^ U-87 MG cells were cultured in 0 and 60 µM honokiol for 7 days. (**A**) Representative image of U-87 MG spheroids. (**B**) Quantification of U-87 MG spheroid numbers. *** *p* < 0.001 vs. control group. (**C**) CD133, Nestin and p-STAT3 levels as determined by western blot analysis. (**D**) Images of cells immunostained for CD133 (red) and Nestin (green). Nuclei are counterstained with DAPI (blue). Scale bar: 20 μm.

Spheroid formation is a typical characteristic of glioma stem cells. Based on the inhibition of CD133 and Nestin by honokiol, we hypothesized that honokiol may inhibit spheroid formation of stem cells. As indicated by the number of spheroids, 60 μM honokiol reduced spheroid formation of U-87 MG stem cells (Figure 8A,B). Western blot analysis also showed reduced protein levels of CD133, Nestin and phosphorylation of STAT3 in stem cell spheroids (Figure 8C). In agreement with the western blot results, immunofluorescence staining demonstrated reduced fluorescence intensity for both CD133 and Nestin in response to 60 μM of honokiol in U-87 MG stem cell spheroids (Figure 8D).

### 2.9. Honokiol Inhibits STAT3 Signaling in Glioma Stem Cells

**Figure 9:**
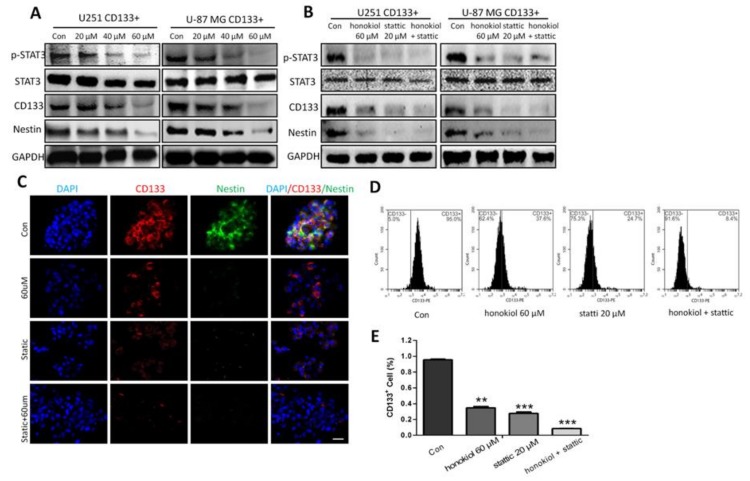
Honokiol down-regulates CD133 and Nestin expression via the JAK/STAT pathway in CD133 immunopositive cells. (**A**) CD133, Nestin and p-STAT3 levels were determined by western blot analysis. (**B**) Administration of 60 μM of honokiol, 20 μM of Stattic and mixture of both reduces p-STAT3, CD133 and Nestin levels. (**C**) Expression of CD133 (red) and Nestin (green) with nuclei counterstained with DAPI (blue). Scale bar: 20 μm. (**D**,**E**) Honokiol, Stattic and the mixture of both decrease numbers of U-87 MG cells immunopositive for CD133 and Nestin as evaluated by flow cytometry. Data are presented as means ± SEM from 3 independent experiments. ** *p* < 0.01 and *** *p* < 0.001 vs. vehicle control group.

Studying the effects of honokiol on STAT3 signaling and stemness, we found that honokiol dose-dependently reduced the phosphorylation of STAT3 in stem cells (Figure 9A). Honokiol also dose-dependently reduced CD133 and Nestin expression, with a maximal inhibition at 60 μM in both glioma stem cells (Figure 9A). We further tested whether honokiol can inhibit the stemness of glioma stem cells via the STAT3 signaling pathway. The results demonstrated that compared with the vehicle control, STAT3, CD133 and Nestin levels were reduced. In comparison to a single application of STAT3 inhibitor Stattic, the combined application of both Stattic and honokiol did not further enhance the inhibitory effect of stattic on STAT3 phosphorylation and levels of CD133 and Nestin (Figure 9B). These results were confirmed by immunofluorescence staining, which showed that the fluorescence intensities for both CD133 and Nestin were reduced (Figure 9C). Flow cytometry results also demonstrated that compared with the vehicle control, the number of CD133 positive U-87 MG cell-derived stem cells was reduced (*p* < 0.01 vs. control for both 60 μM of honokiol and 20 μM of Stattic groups and *p* < 0.001 vs. control for both 60 μM of honokiol and 20 μM of Stattic groups) (Figure 9D,E). The combined results indicate that honokiol inhibits the glioma cell stemness mainly through inhibiting the STAT3 signaling pathway.

### 2.10. Honokiol Reduces Tumor Growth after Xenografting into Zebrafish

**Figure 10:**
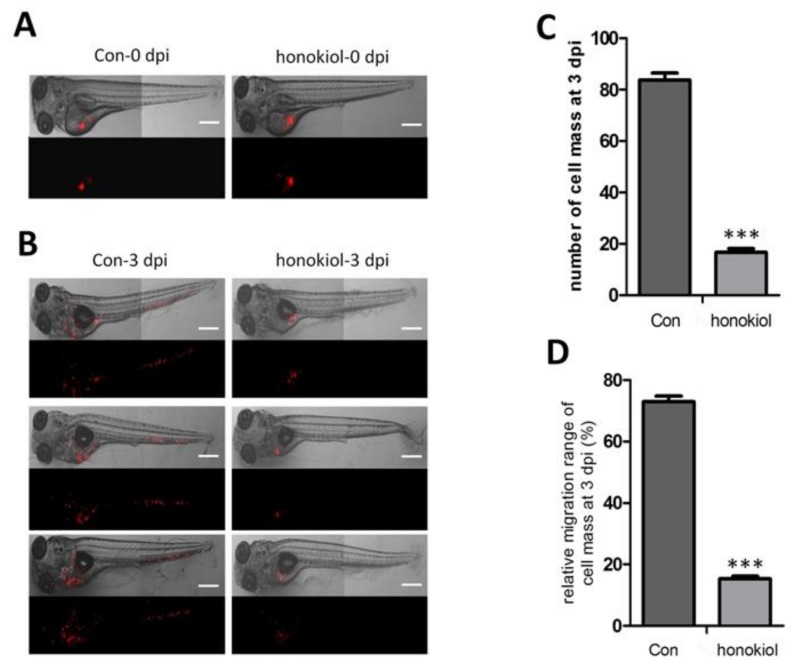
Effects of honokiol in xenografted zebrafish. Two hundred U-87 MG cells treated with honokiol or vehicle control for 48 h were injected into the zebrafish embryo yolk sac. (**A**,**B**) Representative fluorescent images depicting the distribution of transplanted U-87 MG cells in zebrafish at 0 dpi (**A**) and 3 dpi (**B**). Scale bar, 500 μm. (**C**) Number of cell masses in the embryo sac as determined at 3 dpi. (**D**) Quantification of the migration distance of U-87 MG cells relative to zebrafish body length was calculated. Data are presented as means ± SEM from 4 independent experiments. *** *p* < 0.001 vs. control group.

When about 200 CM-DiI labeled U-87 MG cells were injected into the zebrafish yolk sac at 2 dpf and examined by fluorescence microscopy, proliferation and migration of U-87 MG cells at 0 day and 3 days after injection (Figure 10A,B), vehicle-treated cells had migrated from the yolk sac into the whole body including tail and head region, whereas honokiol-treated cells were mainly accumulated in the yolk sac without emigration (Figure 10B). Honokiol treatment reduced the number of cell mass when compared with the vehicle-treated cells at 3 days after injection (*p* < 0.001) (Figure 10C). In addition, honokiol inhibited glioma cell migration, as indicated by the relative cell mass migration at 3 days after injection (*p* < 0.001) (Figure 10D). The combined results indicate that honokiol inhibits glioma cell proliferation and migration in vivo.

### 2.11. Honokiol Reduces Tumor Growth in Nude Mice

**Figure 11:**
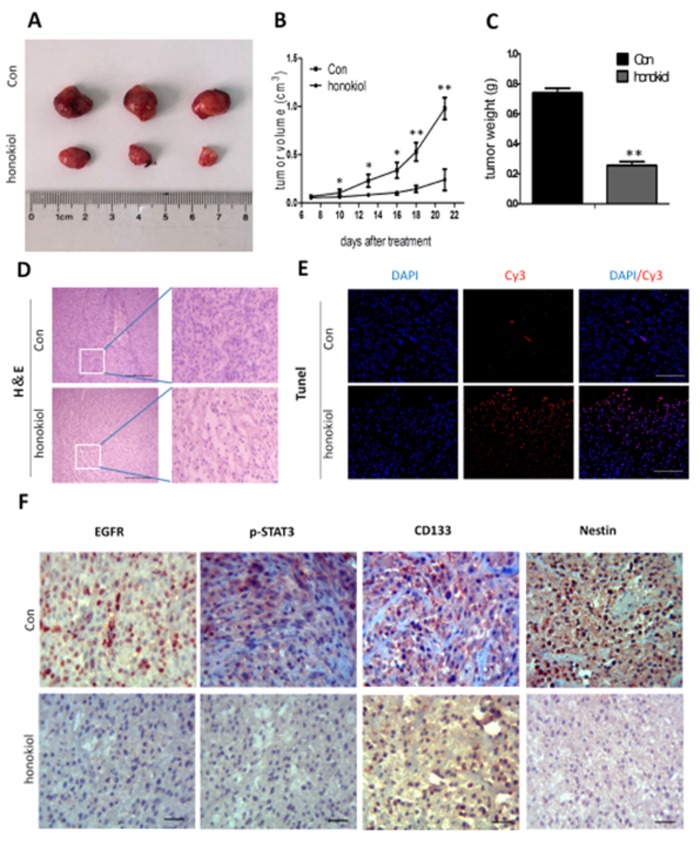
Honokiol inhibits tumor growth in xenografted nude mouse mice. (**A**) Representative images of U-87MG xenografts 21 days after treatment. (**B**) Quantification of tumor volume up to 22 days after grafting ((* *p* < 0.05 and ** *p* < 0.01 vs. vehicle control group). (**C**) Tumor weight at 21 days after treatment. (** *p* < 0.01 vs. vehicle control group). (**D**) Representative H&E stained images 21 days after treatment (**E**) Representative images for TUNEL-positive and DAPI-positive cells in the tumor tissue 21 days after treatment. TUNEL (red) and DAPI (blue). Scale bar: 200 μm. (**F**) Immunohistochemistry of CD133, Nestin, p-STAT3 and EGFR in U-87 MG cell xenografted tumors treated with either vehicle or honokiol. Scale bar: 20 μm.

We then used an in vivo xenograft nude mouse model to further confirm that honokiol reduces cell migration and colony formation. Nude mice were subjected to subcutaneous U-87 MG cell xenografting and then treated with honokiol or vehicle control for 7 days. Thereafter the tumor size was measured at 7, 10, 13, 16, 18, 21 days. Dissected xenografts are shown in Figure 11A, indicating that honokiol treatment inhibits tumor growth at all time points after treatment completion (*p* < 0.05 vs. vehicle control at 10-, 13- and 16-day time points post treatment; *p* < 0.01 vs. vehicle control at 18- and 21-day time points post treatment) (Figure 11B). The average final tumor weight in honokiol-treated mice was lower than that in vehicle-treated mice (*p* < 0.01) (Figure 11C). H&E staining demonstrated that honokiol treatment induced a relatively loosened structure in the xenograft glioblastoma tissue (Figure 11D). Evaluation of the apoptotic cells in the tumor tissue was used using TUNEL assay. Elevated numbers of apoptotic cells were observed in the honokiol-treated glioblastoma tissue (Figure 11E). Furthermore, immunohistochemical analysis demonstrated that EGFR, phosphorylated STAT3, CD133 and Nestin levels are reduced in honokiol-treated tumors compared with the vehicle control (Figure 11F). These data indicate that honokiol induces glioma cell apoptosis in vivo via the regulation of the EGFR-mediated STAT3/JAK signaling pathway.

## 3. Discussion

Glioblastoma accounts for 46.6% of malignant central nervous system tumors in adults [15]. Despite advanced surgery and radio-/chemotherapy developed for treatment of gliomas, the median overall survival is about 14 months [16], with a 5-year survival rate of only 5.5% [15]. In search for an agent that may enhance patient survival, we here show that honokiol ameliorates several indicators of glioma/glioblastoma malignancy, such as migration, invasion, viability and colony formation of stem cells. These findings suggest that honokiol may exert its anticancer functions by inhibition of these parameters via intricate signaling as evidenced in the present study.

Apoptotic signaling, inhibiting tumor progression, promotes cellular self-destruction [17,18]. Anti-apoptotic proteins promote development of many malignant tumors, especially of gliomas [19]. Up-regulation of anti-apoptotic Bcl-2 and Bcl-XL expression contributes to glioblastoma recurrence, in parallel with down-regulation of the pro-apoptotic protein Bax [20]. Down-regulation of Bcl-2 or Bcl-XL results in glioma cell death and sensitization to chemotherapy and radiotherapy [21,22,23]. Honokiol induces apoptosis by enhancing p53 and reducing anti-apoptotic gene expression in human colorectal cancer cells [24].

An important mediator of tumor progression is EGFR, whose expression correlates positively with malignancy and contributes to poor prognosis [25]. EGFR is involved in Jak/STAT, nfb, mTOR and MAPK signaling pathways, thereby influencing cancer cell self-renewal, proliferation, differentiation and migration [26,27,28]. Honokiol can induce mitochondria-dependent and death receptor-mediated apoptosis in multi-drug resistant KB cells by inhibition of EGFR-STAT3 signaling and down-regulation of STAT3 target genes [29]. Honokiol has also been identified as a small molecule agonist of cell adhesion molecule L1 [30]. A L1 fragment (L1-70) is imported from the cell surface via the cytoplasm into mitochondria and increases ATP production by specifically binding to the NDUFV2 enzyme in complex 1 [31]. It remains to be seen how this anti-apoptopic effect of honokiol influences gliomas/glioblastomas. Since the effect of honokiol is highly dose-dependent and was not systematically evaluated in mitochondrial metabolism, it is likely that honokiol, at a high dose, may interfere with this cleavage process, thus partially contributing to mitochondrial malfunction and thereby apoptosis.

Cancer stem cells are the most vital sub-population of cancer cells contributing to drug-resistance and tumor recurrence. Glioma/glioblastoma cells contain a sub-population of glioma initiating cells, which may contribute to chemotherapy-resistance. Previous reports demonstrated that honokiol suppressed metastasis of renal cancer cells via blocking both epithelial-mesenchymal transition and cancer stem cell phenotypes [24]. Honokiol potently inhibits melanoma cell metastasis, in part, through the targeting of melanoma stem cells by suppressing Notch-2 signaling [32]. In agreement with these reports, we found that honokiol reduces levels of EGFR, CD133 and Nestin, indicating that it may attenuate the stemness of glioma stem cells by inhibiting EGFR expression. Thus, a combined application of honokiol and other therapeutic means targeting tumor stem cells may prove effective in treating high-grade glioma.

MMPs are a large family of zinc-dependent endopeptidases that are capable of cleaving multiple extracellular matrix proteins. MMPs can be subdivided into multiple categories depending on their substrate specificity, such as collagenases, stromelysins and gelatinases [33]. Invasive gliomas show MMP2 and MMP9 overexpression and both MMP2 and MMP9 play important roles in infiltrative growth of gliomas [34]. In addition, E-cadherin repression could enhance the generation of cancer stem cells [35]. Increased E-cadherin expression may thus contribute to the attenuation of cancer development. It was also reported that NF-κB promotes migration and invasion of cholangiocarcinoma cells by up-regulating Snail and consequent repression of E-cadherin [36]. We found that honokiol inhibited NF-κB expression, while increasing that of E-cadherin, suggesting that honokiol may inhibit migration and invasion of glioma cells in part by inhibition of MMP2/9 expression, as well as attenuation of the NF-κB-mediated E-cadherin pathway.

The Ras/MAPK/Erk and PI3K/Akt signaling pathways exert crucial roles in cell growth, differentiation, survival and apoptosis in many cancer types [37,38,39,40,41,42]. Honokiol has been shown to inhibit cell proliferation by suppression of the Erk signaling pathway in neuroblastoma cancer cells [43]. Thus, our data imply that honokiol may attenuate proliferation and promote cell apoptosis by inhibition of the PI3K/Akt and MAPK/Erk signaling pathways in glioma cells, which may further inhibit cell migration and colony formation.

To extend these in vitro observations, we performed in vivo experiments to study the effects of honokiol on the growth of glioblastoma U-87 MG xenografts in a zebrafish model [44] and in nude mice. We show that honokiol remarkably suppresses glioblastoma growth in xenografts, in that more apoptotic cells were observed in honokiol-treated glioma tissue. Moreover, EGFR, phosphorylated STAT3, CD133 and Nestin expression was reduced in xenografted glioma tissues in response to honokiol treatment, which was consistent with in vitro results we observed. Honokiol exhibited a profoundly effective inhibition of glioma growth in vivo as observed in the present study. Altogether these results indicate that honokiol inhibits glioma growth and invasion mainly by inhibiting proliferation of glioma stem cells. Unlike intracerebral orthotropic implantation, there is no formation of blood brain barrier (BBB) in the subcutaneous glioma xenograft. This may enhance drug utilization and therapeutic efficacy of honokiol. Intravenous administration of honokiol can effectively attenuate spinal cord lesions in a mouse spinal cord injury model [42], indicating that honokiol may access targeted glioma cells through BBB penetration.

An early report demonstrated that honokiol exerts a preventively anticancer effect by inhibiting metastasis and inducing apoptotic cell death of brain tumor cells [45]. Honokiol dose-dependently inhibited the proliferation of stem-like side population (SP) cells of GBM and strongly reversed Temozolomide (TMZ) resistance by inducing apoptosis in GBM8401 cells [46]. It is recently implicated that honokiol can enhance TMZ-induced apoptotic insults to glioma cells via an intrinsic mitochondrion-dependent mechanism [47]. Our results advanced these reports by demonstrating that honokiol may exert these auxiliary anti-cancer roles via an EGFR-mediated JAK-STAT3 signaling pathway. It remains elucidation whether honokiol can function like N6-isopentenyladenosine (iPA), which has been reported to display an immune-mediated antitumor activity [48].

In summary, with the combined application of both in vitro and vi vivo experiments, we advanced these reports by further clarifying that he traditional Chinese herb honokiol can inhibit glioma stem cell proliferation by inducing the apoptosis of glioma stem cells via EGFR-mediated JAK-STAT3 signaling pathway. Honokiol contributes to EMT inhibition by increasing the expression of E-Cadherin. Honokiol also Inhibits glioma/glioblastoma progression by targeting EGFR. The combined observations indicate that honokiol may represent a novel therapy for malignant glioma.

## 4. Materials and Methods

### 4.1. Cells and Reagents

U251 human glioma and U-87 MG human glioblastoma cell lines were purchased from the Chinese Type Culture Collection (CTCC, Shanghai, China) and were maintained in Dulbecco’s modified Eagle’s medium low Glucose (DMEM, SH30021.01, Thermo Scientific HyClone, Beijing, China) supplemented with 50 U/mL of a penicillin/streptomycin mixture (Solarbio Biotech, Beijing, China) and 10% fetal bovine serum (Sijiqing Biotech, Hangzhou, China). CD133^+^ cells were maintained in DMEM-F12 medium supplemented with 20 ng/ml of recombinant human epidermal growth factor (EGF) (10605-HNAE) and recombinant human basic fibroblast growth factor (bFGF) (10014-HNAE) and 2% B27 supplement (Gibco, Grand Island, NY, USA) and 1% penicillin/streptomycin solution (Solarbio Biotech). The cells were routinely grown in 60-cm^2^ cell culture plates (Corning Incorporated, Corning, NY, USA) at 37 °C in a humidified atmosphere with 5% carbon dioxide (CO_2_). Honokiol and Stattic were purchased from Santa Cruz Biotechnology (Santa Cruz, CA, USA). MTT and TUNEL assay kits were purchased from Beyotime Biotechnology (Haimen, Jiangsu, China).

### 4.2. MTT Assay

U251 and U-87 MG cells were seeded into a 96-well plate at a density of 3 × 10^3^ cells per well. After overnight incubation, the culture medium was aspirated and honokiol’s effect on cell viability cells was measured at doses ranging from 0 to 60 μM in complete culture medium after incubation times of 24 and 48 h in 0.1% DMSO, with a vehicle control also comprising 0.1% DMSO. Then, 10 µl of MTT (5 mg/ml; Beyotime) was added to each well and cells were further cultured for 4 h. After removal of the culture medium, 100 µl of DMSO was added. The absorbance was measured at a wavelength of 490 nm in an ELISA plate reader (Infinite M1000, Tecan, Switzerland). Cell survival was determined using the formula: Survival rate (%) = mean OD_treated groups_/OD_vehicle control group_. The half-maximal inhibitory concentration (IC50) at 48 h was calculated, with the survival of vehicle-treated cells being set at 100%. Results are from three independent experiments.

### 4.3. Scratch Assay

U-87 MG cells were seeded at a density of 5 × 10^4^ cells per well into a 96-well plate in complete cell culture medium until confluency. After treatment with different concentrations of honokiol, the monolayer was scratched with a 10 μL plastic pipette tip to create a space. The space width was then examined under a phase-contrast microscope at × 100 magnification (Olympus IX51, Tokyo, Japan) after 0, 24 and 48 h of incubation. Photographs of at least three random fields were taken and cell spreading into the space was determined by measuring the gap width. Results are from at least three independent experiments.

### 4.4. Invasion Assay

For this assay, U251 and U-87 MG cells were incubated with 0, 20, 40, 60 μM honokiol for 48 h. Then, 1 × 10^4^ cells in 100 μL medium without fetal bovine serum were seeded onto a polycarbonate membrane insert with 8 μm pores (underside precoated with fibronectin) in a Transwell Apparatus (Costar, Corning) and seeded onto the upper side of the membrane pre-coated with Matrigel (Becton Dickinson Biosciences, Palo Alto, CA, USA). The lower chamber was loaded with 500 μL medium supplemented with 10% fetal bovine serum. After incubation for 12 h at 37 °C in a 5% CO_2_ atmosphere, the membrane was washed with PBS and cells on the membrane top surface were removed with a cotton swab. Cells adhering to the underside were fixed with 4% formaldehyde, stained with crystal violet solution and counted under a microscope. Results are from three independent experiments.

### 4.5. Colony Formation Assay

U251 and U-87 MG cells (1500 cells/well) were seeded into a 24-well plate. After treatment with 0, 20, 40, 60 μM honokiol at 37 °C for 10 days, the colonies were fixed with methanol for 20 min, stained with 0.1% crystal violet and visualized under a phase-contrast light microscope (Olympus IX51). A cluster of more than 50 cells was identified as a colony. Results are from three independent experiments.

### 4.6. Flow Cytometric Assay of Apoptosis

U251 and U-87 MG cells were seeded at a density of 5 × 10^5^ cells per well into 6-well plates in complete culture medium. After overnight incubation, the culture medium was removed and the cells were treated with honokiol at doses ranging from 0 to 60 µM in complete culture medium. Cells were then further cultured for 24 h and 48 h. Apoptosis was evaluated with the Annexin V-EGFP/PI Apoptosis Kit according to the manufacturer’s protocol (Beyotime) using the Accuri C6 flow Cytometer (Becton Dickson Biosciences). Annexin V-EGFP/PI was excited with 495 and 535 nm lasers and signals were detected using 530/30 and 585/40 band pass filters, respectively.

### 4.7. Assay for CD133-Positive Cells

U-87 MG cells were seeded at a density of 5 × 10^5^ cells per well into 6-well plates, incubated with 0 to 60 µM honokiol in DMEM-F12 medium supplemented with 20 ng/ml of recombinant human EGF (Sigma-Aldrich, St. Louis, MO, USA) and recombinant human bFGF (Sigma) and 2% B27 supplement (Invitrogen) and 1% penicillin/streptomycin solution (Solarbio Biotech). After 7 days, cultures were evaluated for CD133 immunopositive stem cells by flow cytometry (see Section 4.8).

### 4.8. Flow Cytometric Cell Sorting

U251 and U-87 MG cells were trypsinized and washed with PBS three times for 3 min each, followed by a wash in DMEM-F12 and FACS buffer (PBS containing 1% BSA). Then, 10^7^ cells were resuspended in 2 ml FACS buffer on ice and 10 μL of anti-CD133-PE antiserum (Miltenyi, Cat. No. 130-090-853; Bergisch Gladbach, Germany) was added. Cells were then incubated on ice for 15 min in the dark, pelleted by centrifugation at 1000× *g* for 5 min at 4 °C and washed once with FACS buffer. Stained cells were sorted with the FACSII (Becton and Dickinson Biosciences). Cell debris were gated out using a forward scatter/sideward scatter dot plot. CD133-negative and CD133-positive cell populations were collected.

### 4.9. Flow Cytometry of CD133^+^ Cells

U251 and U-87 MG CD133^+^ cells were cultured at a density of 5 × 10^5^ cells per well into 6-well plates in DMEM-F12 medium supplemented with 20 ng/ml of each recombinant human EGF (Sigma) and recombinant human bFGF (Sigma) and 2% B27 supplement (Invitrogen) and 1% penicillin/streptomycin solution (Solarbio Biotech). Effects of honokiol or Stattic, as well as their combined effects on the stemness of CD133^+^ cells were evaluated by treatment with 60 µM honokiol, 20 µM Stattic and mixture of 60 µM honokiol and 20 µM Stattic in DMEM-F12. Cells were then further cultured for 48 h. Thereafter cells were washed with PBS three times for 3 min each and washed in DMEM-F12 and FACS buffer (PBS containing 1% BSA). 10^7^ cells were resuspended in 2 ml FACS buffer on ice. Ten µl of anti-CD133-PE antiserum was added and cells were incubated for 15 min on ice in the dark. Cells were pelleted and washed once with FACS buffer. Stained cells were measured using the Accuri C6 Cytometer (Becton Dickinson Biosciences).

### 4.10. Immunofluorescence Staining

U251 and U-87 MG cells were seeded into glass coverslip in a 24-well plate at a density of 5 × 10^4^ cells per well. After overnight incubation, the culture medium was removed and the cells were incubated with honokiol at doses ranging from 0 to 60 µM in complete culture medium and cells were further cultured for 48 h. Fixed U251 and U-87 MG cells were incubated with anti-EGFR (ErbB1) antibody (1:200; Cat. No. 66455-1-Ig, Proteintech, Rosemont, IL, USA) overnight at 4 °C. After washing in PBS three times for 5 min each, the samples were incubated at room temperature with FITC-conjugated goat anti-mouse IgG antibody (1:500; Jackson ImmunoResearch, West Grove, PA, USA) for 60 min at room temperature. The cells were then stained with 4′,6-diamidino-2-phenylindole (DAPI) and mounted using anti-fade mounting solution (Beyotime).

### 4.11. Immunofluorescence Staining of CD133^+^ Cells

CD133^+^ cells were seeded onto glass coverslips coated with poly-L-lysine (Sigma-Aldrich). After 12 h they were incubated with honokiol or Stattic at doses of 0, 20 and 60 µM for 48 h in DMEM/F12 culture medium. Cells were fixed and incubated with antibodies against CD133/1 (1:50; Cat. No. 18470-1-AP, Proteintech) and Nestin (1:50; Cat. No. 66259-1-Ig, Proteintech) overnight at 4 °C After washing in PBS three times for 5 min each, the samples were incubated at room temperature with goat anti-mouse fluorescein (FITC)–conjugated antibody (1:500; Jackson ImmunoResearch) and anti-rabbit secondary antibody conjugated to Dylight^TM^ 594 (1:500; Jackson ImmunoResearch) for 60 min. The cells were then stained with DAPI and mounted using anti-fade mounting solution (Beyotime).

### 4.12. Western Blot Analysis

Cell lysates were heated at 100 °C in sample loading buffer (0.125 mol/L Tris-HCl, pH 6.8, 20% glycerol, 10% SDS, 0.1% bromophenol blue and 5% β-mercaptoethanol), resolved by 10% SDS polyacrylamide gel electrophoresis and electroblotted onto polyvinylidene difluoride membranes (PVDF, Merck KGaA, Darmstadt, Germany). Non-specific protein binding sites were blocked with 5% BSA (Cat. No. #A8020, Solarbio, Beijing, China) diluted in Tris-buffered saline buffer containing 0.05% Tween-20 (TBST, pH 7.4). The membranes were incubated with antibodies specific for ERK (MK1; 1:1000; Cat. No. sc-135900, this and the following antibodies were purchased from Santa Cruz Biotechnology and the dilutions and catalog numbers are indicated for each antibody), AKT (G-5; 1:1000; Cat. No. sc-53373), p-ERK (E-4; 1:1000; Cat. No. sc-7383), p-AKT (11E6; 1:1000; Cat. No. sc-81433), Bcl-2 (C-2; 1:1000; Cat. No. sc-7382), Bax (C-19; 1:1000; Cat. No. sc-526), STAT3 (H-190; 1:1000; Cat. No. sc-7179), p-STAT3 (Ser 727; 1:1000; Cat. No. sc-8001-R), NFkB (A; 1:1000; Cat. No. sc-109), MMP9 (C-20; 1:1000; Cat. No. sc-6840), MMP2 (K-20; 1:1000; Cat. No. sc-8835), beta-catenin (6F9; 1:1000; Cat. No. sc-53484), E-cadherin (H-108; 1:1000; Cat. No. sc-7870) and GAPDH (2E3-2E10; 1:1000; Cat. No. sc-293335). In the following used antibodies are both against pro- and cleaved caspase-3 (1:500; Cat. No. BM3257, Boster; Wuhan, China), CD133 (1:1000; Cat. No. 18470-1-AP, Proteintech); Nestin (1:1000; Cat. No. 66259-1-Ig, Proteintech); EGFR (1:1000; Cat. No. 66455-1-Ig, Proteintech). They were also incubated overnight at 4 °C. After 3 washes for 5 min each, HRP-conjugated goat anti-mouse (Cat. No. BA1051, Boster) and goat anti-rabbit (Cat. No. BA1055, Boster) secondary antibodies (1:1000 for both; Boster) diluted in 5% BSA in TBST were applied, followed by 3 washes with TBST for 5 min each at room temperature. Signal intensities were quantified using Image J software (https://imagej.nih.gov/ij/) as the average densitometry multiplied by the area (measured as the number of pixels).

### 4.13. Zebrafish Xenografting

Zebrafish were maintained according to standard procedures [49]. Wild type zebrafish were purchased from Huiyuan Aquatic Animals Company (Shantou, Guangdong, China). Twenty-four h post fertilization (dpf), larvae were treated with PTU (1-phenyl 2-thiourea, 0.2 mM) to remain transparent for microscopic analysis. Two dpf larvae were anaesthetized with 0.003% tricaine (Sigma) and positioned on a 10 cm dish coated with 3% agarose. Cell suspensions were injected by a micromanipulator (LPP01-100, LongerPump, Wuhan, China) equipped with borosilicate glass capillary needles (1.5 mm in outer diameter and 0.86 mm in inner diameter) which were made by a P-97 micropipette puller (Sutter Instrument Company, Novato, CA, USA). Approximately 200 cells in a volume of 10 μL were injected into the yolk sac of larvae, which were then maintained in E3 medium/PTU for 1 h at 28 °C and 32 °C. Images were captured at 0- and 3-day time points post injection using a Zeiss fluorescence microscope (Axio Observer A1, Oberkochen, Germany). All experiments were approved by the local authorities (ethical code: SUMC2018-125, permission date: 30 June 2018).

### 4.14. Nude Mouse Xenografting

Four-week-old nude female mice were purchased from the Model Animal Research Center of Nanjing University and maintained at the animal facility of Shantou University Medical College for 1 week prior to experimental use. U-87 MG cells (5 × 10^5^ cells/mouse) were subcutaneously injected into the right flank near the upper extremity. After the tumors had reached a volume of 2 mm^3^, mice were randomized into two groups (*n* = 3 per group). Then, either honokiol (100 mg/kg/day) or DMSO vehicle control were intraperitoneally injected daily for 7 days. The tumor size was measured using a Vernier caliper (BST-01601, BESTIR, Foshan, Guangdong, China) at days 7, 10, 13, 16, 18 and 21 after treatment. Tumor volume was estimated by the formula (L × S^2^/2, L: largest diameter and S: smallest diameter). Mice were sacrificed and tumors were dissected out and weighed using an electronic balance (ME104, Mettler Toledo, Shanghai, China). All experiments were approved by the Institutional Animal Care and Use Committee of Shantou University Medical College (ethical code: SUMC2018-119, permission date: 30 May 2018).

### 4.15. H & E Staining and Immunofluorescence Staining of Xenografted Tumor Cells

Tumors were cryosectioned at 4 μm for hematoxylin/eosin staining as described [50]. For estimation of apoptosis, xenograft tumor tissues were evaluated by both DAPI staining and terminal deoxynucleotidyl transferase (TdT) deoxyuridine 5-triphosphate dUTP Nick-End Labeling (TUNEL) staining (Beyotime). Sections were washed using PBS and incubated with TF3-dUTP and DAPI for 60 min at 37 °C. Both TUNEL-positive and Cy3 (Cyanine 3)-positive cells were inspected with a fluorescence microscope (Axio Imager A2, Oberkochen, Germany). Photographs of at least five random fluorescent fields were taken.

### 4.16. Immunohistochemistry

Immunohistochemical staining was performed for evaluation of expression and localization of EGFR, pSTAT3, CD133 and Nestin. In brief, deparaffinized sections were rehydrated through a graded series of ethanol to phosphate buffered saline (PBS). Antigen retrieval was performed and endogenous peroxidase clearance was performed by incubation with 3% H_2_O_2_. Sections were then blocked with 10% normal goat serum (AR009, Boster) for 30 minutes and individually incubated with rabbit anti-CD133 (1:200; Cat. No. 18470-1-AP, Proteintech), mouse anti-Nestin (1:200; 66259-1-Ig, Proteintech), mouse anti-EGFR (1:1000; 66455-1-Ig, Proteintech), rabbit anti-p-STAT3 (1:200; Cat. No. sc-8001-R, Santa Cruz) antibodies. The antigen-antibody complexes were visualized using the AEC method (Zhongshan Goldbridge Biotechnology, Beijing, China) and counterstained with hematoxylin (Zhongshan Goldbridge Biotechnology). Immunohistochemical staining was analyzed using a Jiangnan light microscope DN-10B (Jiangnan, Nanjing, Jiangsu, China).

### 4.17. Statistics

Statistical analyses were performed using SPSS (Statistical Package for the Social Sciences) 19.0 software (SPSS, Chicago, IL, USA). The intensities were quantified using Image J software. Data are expressed as means ± SEM of at least 3 to 4 independent experiments and compared using either two-tailed independent Student’s *t*-test or one-way ANOVA with Tukey’s *post-hoc* test for multiple comparisons. Differences were considered to be significant at *p* < 0.05.

## 5. Conclusions

The traditional Chinese herb honokiol inhibits glioma/glioblastoma cell migration and proliferation mainly by attenuating the generation of glioma/glioblastoma stem cell-like cells via JAK-STAT3 signaling. Honokiol also Inhibits glioma/glioblastoma progression by targeting epidermal growth factor receptor. The combined observations indicate that honokiol may represent a novel therapy for malignant glioma.
